# Eukaryotic Initiation Factor 4H Is under Transcriptional Control of p65/NF-κB

**DOI:** 10.1371/journal.pone.0066087

**Published:** 2013-06-11

**Authors:** Giuseppe Fiume, Annalisa Rossi, Annamaria de Laurentiis, Cristina Falcone, Antonio Pisano, Eleonora Vecchio, Marilena Pontoriero, Iris Scala, Annarita Scialdone, Francesca Fasanella Masci, Selena Mimmi, Camillo Palmieri, Giuseppe Scala, Ileana Quinto

**Affiliations:** 1 Department of Experimental and Clinical Medicine, University of Catanzaro “Magna Graecia”, Catanzaro, Italy; 2 Department of Pediatrics, University of Naples “Federico II”, Naples, Italy; 3 Department of Molecular Medicine and Medical Biotechnology, University of Naples “Federico II”, Naples, Italy; NIGMS, NIH, United States of America

## Abstract

Protein synthesis is mainly regulated at the initiation step, allowing the fast, reversible and spatial control of gene expression. Initiation of protein synthesis requires at least 13 translation initiation factors to assemble the 80S ribosomal initiation complex. Loss of translation control may result in cell malignant transformation. Here, we asked whether translational initiation factors could be regulated by NF-κB transcription factor, a major regulator of genes involved in cell proliferation, survival, and inflammatory response. We show that the p65 subunit of NF-κB activates the transcription of eIF4H gene, which is the regulatory subunit of eIF4A, the most relevant RNA helicase in translation initiation. The p65-dependent transcriptional activation of eIF4H increased the eIF4H protein content augmenting the rate of global protein synthesis. In this context, our results provide novel insights into protein synthesis regulation in response to NF-κB activation signalling, suggesting a transcription-translation coupled mechanism of control.

## Introduction

Protein synthesis is timely regulated at the step of translation initiation. Loss of translation control may lead to cell malignant transformation as consequence of increased rate of protein synthesis and translation activation of mRNA species that are relevant for cell proliferation and survival.

Initiation of protein synthesis of most eukaryotic mRNAs is m^7^G-cap-dependent and requires at least 13 eukaryotic initiation factors (eIFs) [1], [2]. The heterotrimeric protein complex eIF4F that includes eIF4A, eIF4E and eIF4G, binds to the m^7^G-cap of mRNA through eIF4E, and to the poly (A)-binding protein through the scaffolding adapter eIF4G; these protein-mRNA interactions allow the recruitment, circularization and activation of the bound mRNA. The multiprotein complex, including eIF3, eIF1, eIF1A, eIF5 and eIF2–GTP–Met-tRNA_Met_, binds to the 40S ribosomal subunit to generate the 43S pre-initiation complex. Then, interaction of eIF4G with eIF3 recruits the 43S ribosomal complex to mRNA in order to generate the pre-initiation complex (PIC) that scans the mRNA in 5′-3′ direction towards the initiation codon, where PIC associates with the 60S ribosomal subunit to constitute the 80S initiation complex. Sliding of PIC along the mRNA requires the ATP-dependent RNA helicase eIF4A, which unwinds secondary structures that occur at the 5′-untranslated region of more than half of human mRNAs, and is required for scanning processivity of the 40S ribosomal subunit toward the initiation codon [2]. The homologous RNA binding proteins eIF4B and eIF4H associate with eIF4A in a mutually exclusive manner, and stimulate the eIF4A helicase activity [3], [4].

Cell signalling timely controls protein synthesis at the translation initiation step. In fact, mitogens activate the MAPK and PI3K/mTOR signalling cascades that ultimately promote protein synthesis by post-translational modifications of eIFs and their regulators. In particular, MAPK pathway promotes the 5′cap-dependent translation through eIF4E phosphorylation [5]; the PI3K/mTOR pathway promotes the 5′cap-dependent translation through phosphorylation of the 4E-BP1 inhibitor of eIF4E [6] and the PDCD4 inhibitor of eIF4A [7]. Moreover, both MAPK and PI3K/mTOR pathways cause eIF4B phosphorylation, which promotes eIF4B association with eIF4A, stimulating the helicase activity [8]. On the opposite side, nutrient deprivation, apoptosis and viral infections inhibit translation initiation as a consequence of activation of stress-regulated protein kinases and IFN-induced double-stranded RNA-activated protein kinase (PKR) that phosphorylate the eIF2 alpha subunit, preventing the assembly of the eIF2/GTP/Met-tRNA complex [9].

As additional mechanism of translation control, initiation factors can be regulated at the transcriptional level determining their cellular protein content. Enhanced expression of some eIFs leads to increased protein synthesis [1]. Over-expression of eIF4E, eIF4A1, eIF2α and p40 or p110 subunits of eIF3 was observed in tumors, suggesting that neoplastic transformation was a consequence of increased translation of relevant mRNA species for cell proliferation and survival [10], [11]. Consistently, anti-sense RNAs targeting eIF4E, eIF4A and eIF4B decreased the proliferation rate of tumor cells and their ability to grow in soft agar [12], [13].

NF-κB transcription factors play a major role as regulators of genes involved in cell proliferation, survival, and inflammatory response [14]–[16]. The NF-κB family includes RelA/p65, c-Rel, RelB, p50 and p52 proteins that share a highly conserved 300-amino acid Rel homology domain (RHD), which is required to homo- or hetero-dimerize, and to bind to DNA enhancer. The transcriptional activity of the NF-κB complex depends on subunits composition since C-terminal unrelated transcriptional activation domains are exclusively present in p65, RelB and c-Rel subunits. Inhibitors of NF-κB (IκB) associate with the NF-κB complex and interfere with its binding to DNA. The canonical pathway of NF-κB activation leads to IκB kinase (IKK)-mediated phosphorylation of IκB at specific serine residues that target IκB to ubiquitination followed by 26S proteasome-dependent degradation; this event releases the NF-κB complex for transcriptional activation. IκB-α is the most abundant and ubiquitous inhibitor of NF-κB family [17], and acts as constitutive repressor of NF-κB and tumor suppressor when mutated to the proteolysis-resistant IκB-α S32/36A form [18], [19].

In this study, we addressed the question of whether NF-κB could affect the eIFs gene expression. We found that the p65 subunit of NF-κB activates the transcription of eIF4H, a regulatory subunit of the RNA helicase eIF4A. The p65-dependent transcriptional activation of eIF4H caused an increase of both eIF4H protein content and protein synthesis rate. The regulation of eIF4H gene by p65 provides new insights into protein synthesis regulation in response to NF-κB activation, suggesting a transcription-translation coupled mechanism of protein synthesis control.

## Results

### eIF4H Gene Expression is Activated by p65 Subunit of NF-κB

**Figure 1:**
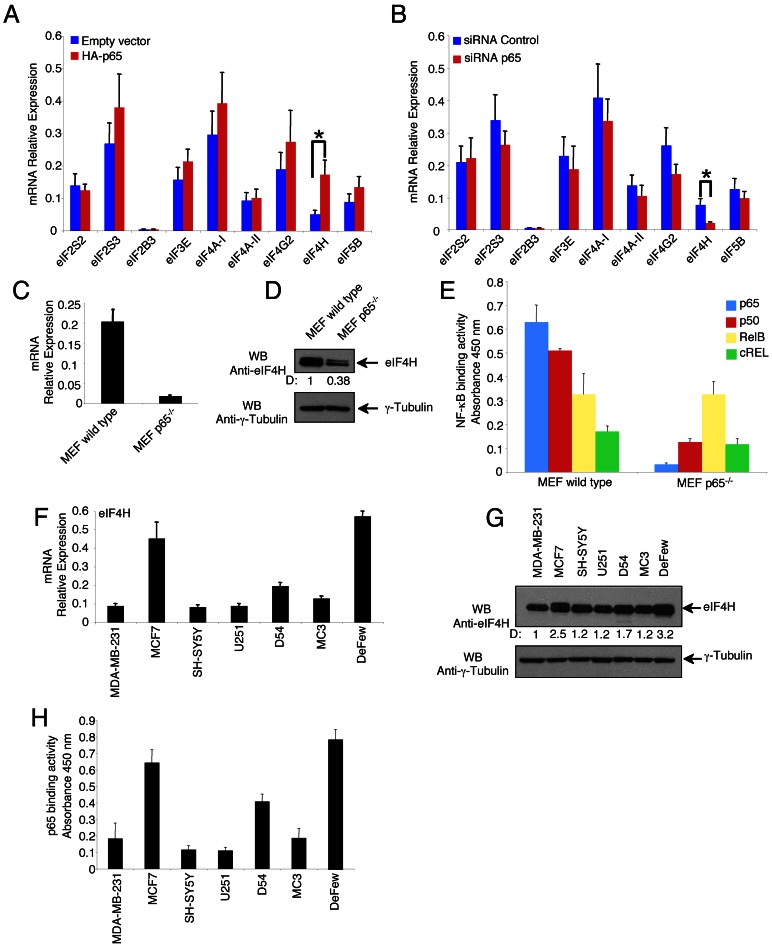
p65-dependent transcriptional activation of eIF4H. (A) HeLa cells (5×10^6^) were transfected with pRc/CMV-p65 or pRc/CMV-empty vector (5 µg). Forty-eight hours post-transfection, total RNA was extracted and analysed by qRT-PCR to evaluate the expression of the indicated eIF genes. Values (mean ± SD, n = 5) are shown. The asterisk indicates a statistically significant difference between pRc/CMV-p65 and empty vector according to the Student's *t*-test (*p*≤0.01). (B) HeLa cells (5×10^6^) were transfected with siRNA control or siRNA p65 (200 pmol). Forty-eight hours post-transfection, total RNA was extracted and analysed by qRT-PCR for the expression of the indicated eIF genes. Values (mean ± SD, n = 5) are shown. The asterisk indicates a statistically significant difference between siRNA p65 and siRNA control according to the Student's *t*-test (*p* ≤0.01). (C) Wild type and p65^−/−^ MEFs (3×10^5^) were lysed, and total RNA was analysed by qRT-PCR for the expression of eIF4H gene. (D) Total cell extracts (20µg) of wild type and p65^−/−^ MEFs (3×10^5^) were separated by 12% SDS-PAGE and analysed by western blotting using anti-eIF4H, or anti-γ-Tubulin antibodies. Densitometry values (D) of the bands were expressed as fold increase above the wild type taken as 1. (E) Nuclear extracts of wild type and p65^−/−^ MEFs (5×10^6^ cells) were analysed for the binding activity of the indicated NF-κB subunits to the NF-κB double-stranded oligonucleotide, as measured by ELISA EMSA using the NF-κB Transcription Factor ELISA assay kit (Cayman). (F) Total RNA from tumour cell lines (MDA-MB-231, MCF-7, SH-SY5Y, U251, D54, MC3, DeFew) (3×10^5^ cells) was analysed by qRT-PCR for the expression of eIF4H gene. (G) Whole protein cell extracts (20µg) of the indicated tumour cell lines were separated by 12% SDS–PAGE and analysed by western blotting using anti-eIF4H, or anti-γ-Tubulin antibodies. Densitometry values (D) of the bands were expressed as fold increase above the MDA-MB-231 cells taken as 1. (H) Nuclear extracts of the indicated tumor cell lines (5×10^6^ cells) were analysed for the p65 binding to the NF-κB double-stranded oligonucleotide, using the NF-κB Transcription Factor ELISA assay kit (Cayman).

Computational analysis using Jaspar (http://jaspar.genereg.net/) and TFSEARCH (http://www.cbrc.jp/research/db/TFSEARCH.html) as bioinformatics tool predicted putative NF-κB consensus sites in the promoter regions of several eIFs, including eIF2S2, eIF2S3, eIF2B3, eIF3E, eIF4A1, eIF4A2, eIF4G2, eIF4H (Table 1). To check whether the predicted NF-κB consensus sites acted as enhancers in response to NF-κB, HeLa cells were transfected with an expression vector of the p65 transcriptional subunit of NF-κB, or empty vector, and forty-eight hours later the eIF genes transcripts were measured by real time quantitative PCR (qRT-PCR). p65 significantly enhanced the eIF4H transcription, without affecting the expression of the other eIFs (Figure 1A). Consistently, p65 RNA interference significantly reduced the basal expression level of eIF4H, without affecting the other eIFs (Figure 1B). Other subunits of NF-κB, such as p50, c-Rel and RelB, did not significantly affect the expression of eIF4H gene, or other eIFs (Figure S1). These results suggest that eIF4H was the only NF-κB-responsive gene among the analysed eIFs, and p65 was the main NF-κB transcriptional subunit acting on the eIF4H promoter. The p65-dependent expression of eIF4H was confirmed in wild type and p65^−/−^ MEFs, where the eIF4H gene expression and eIF4H protein level were both lowered in the absence of p65 activity (Figure 1C, D, E). Further, the eIF4H gene expression and protein content correlated with the level of p65 DNA binding activity in different cancer cell lines, such as breast cancer (MDA-MB-231, MCF7), neuroblastoma (SH-SY5Y), glioblastoma (U251, D54) and B-cell lymphoma (MC3, DeFew), supporting the role of p65 as transcriptional activator of eIF4H in different cellular contexts (Figure 1F - H).

**Table 1:** Bioinformatics-based prediction of NF-κB sites in eIFH promoters.

	Gene Name	PutativeNF-κB site(s) byJASPAR	PutativeNF-κB site(s) byTFSEARCH	Position on promoter	NF-κB consensussequence
**Core Initiation Factors**	EIF1	0	0		
	EIF1A	0	0		
	EIF2S1(eIF2-alpha)	0	0		
	EIF2S2(eIF2-beta)	1	0	−684;−675	CGGTGTTTCC
	eIF2S3(eIF2-gamma)	1	1	−107; −98	TGGAGTTTCC
	eIF2B1	0	0		
	eIF2B2	0	0		
	eIF2B3	1	1	−730; −721	GGGACTTCCT
	eIF2B4	0	0		
	EIF3A	0	0		
	EIF3B	0	0		
	EIF3E	1	0	−98; −89	GGGGTTTACC
	EIF3F	0	0		
	EIF3G	0	0		
	EIF3H	0	0		
	EIF3I	0	0		
	EIF3J	0	0		
	EIF3K	0	0		
	EIFS8	0	0		
	EIF4A1	1	1	−418; −408	GAGGATTCCCC
	EIF4A2	2	2	−362; −353−281; −272	GGGGCTTCCC;CGGGGTTTCC
	EIF4A3	0	0		
	EIF4B	0	0		
	EIF4E	0	0		
	EIF4G1	0	0		
	EIF4G2	1	0	−671; −662	CGGACATTCC
	EIF4G3	0	0		
	EIF4H	2	2	−253; −244−210; −201	GGGAGTTCCC;CGGCCTTTCC
	EIF5	0	0		
	EIF5B	1	0	−238; −229	CGGGTTTTCC

Jaspar- and TFSEARCH-based computational analysis predicted putative NF-κB enhancers in the promoter regions of core eukaryotic translation initiation factors.

### Tumour Necrosis Factor-α Activates the eIF4H Gene Expression through p65 Recruitment to eIF4H Promoter

**Figure 2:**
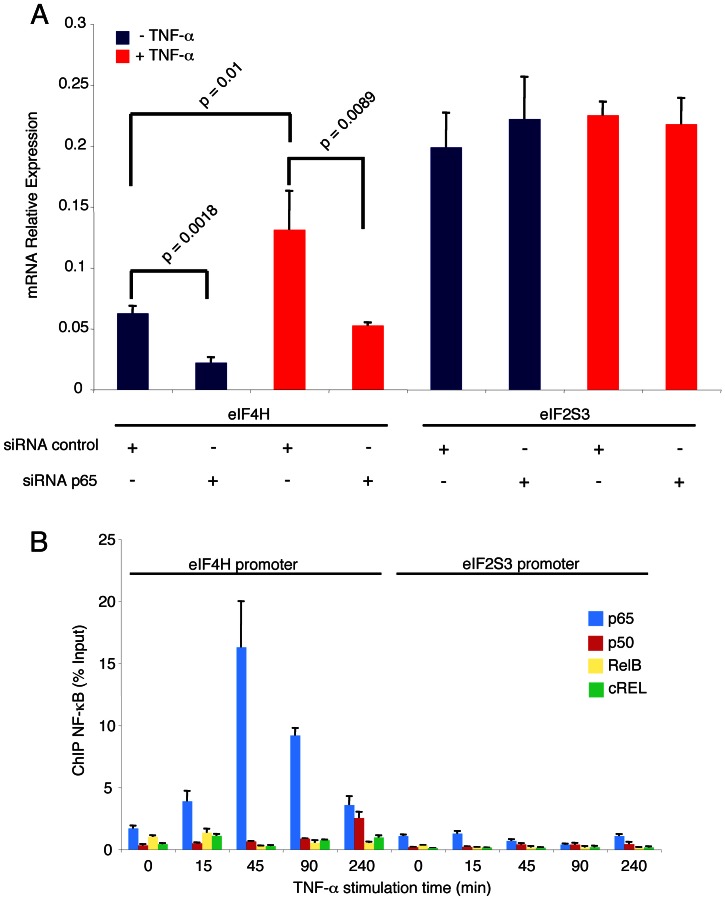
TNF-α induces the recruitment of p65 to eIF4H promoter. (A) HeLa cells (5×10^6^) were transfected with siRNA control or siRNA p65 (200 pmol). Forty-eight hours post-transfection, cells were 45 min-treated with TNF-α (20 ng/mL), or left untreated. Total RNA was extracted and analysed by qRT-PCR to measure the expression of eIF4H and eIF2S3. Values (mean ± SD, n = 3) are shown. Statistically significant differences between the samples are shown according to Student's *t*-test (p≤0.01). (B) HeLa cells (3×10^7^) were treated with TNF-α (20 ng/mL) for the indicated time, or left untreated. Chromatin was immunoprecipitated with anti-p65, anti-p50, anti-RelB, anti-c-Rel or IgG, and ChIP eluates were analysed by qRT-PCR.

We asked whether the NF-κB activation signalling could modulate the expression of eIF4H gene through p65. To this end, HeLa cells were transfected with siRNA p65, or siRNA control, and then stimulated with tumour necrosis factor-α (TNF-α), a well-known NF-κB inducer, or left un-stimulated; forty-eight hours later, total RNA was analysed by qRT-PCR to measure the eIF4H gene expression. TNF-α induced two-fold increase of eIF4H transcripts, and this induction was abolished upon p65 RNA interference (Figure 2A). In the same experimental conditions, eIF2S3, a p65-unresponsive eIF gene (Figure 1A, B), was unaffected by TNF-α treatment, as well as by p65 RNA interference (Figure 2A).

To further investigate the NF-κB-dependent regulation of eIF4H gene, the occupancy of eIF4H promoter by NF-κB transcription factors was analysed by chromatin immunoprecipitation (ChIP) in HeLa cells, with or without TNF-α stimulation. Consistently with p65-dependent transcriptional activation of eIF4H gene, TNF-α increased the recruitment of p65 to the eIF4H promoter at 15 min post-treatment, with a peak at 45 min, followed by decrease within 240 min (Figure 2B). The other NF-κB transcriptional subunits, cRel and RelB, were not recruited to the eIF4H promoter, accordingly with their lack of effect on eIF4H transcription (Figure S1). Instead, the p50 subunit, which lacks the transcriptional activation domain, was recruited at a late time point post-stimulation (240 min), suggesting that p50 homodimers were replacing p65 homodimers to repress transcription (Figure 2B). As control, TNF-α did not induce the recruitment of NF-κB subunits to the NF-κB-unresponsive eIF2S2 promoter (Figure 2B). Altogether these results indicated that the transcriptional activation of eIF4H by TNF-α was mediated by p65/NF-κB; these findings were consistent with eIF4H transcriptional activation by transfected p65, and eIF4H down-regulation by p65 RNA interference (Figure 1A, B).

### p65 Increases the Cellular eIF4H Protein Content

**Figure 3:**
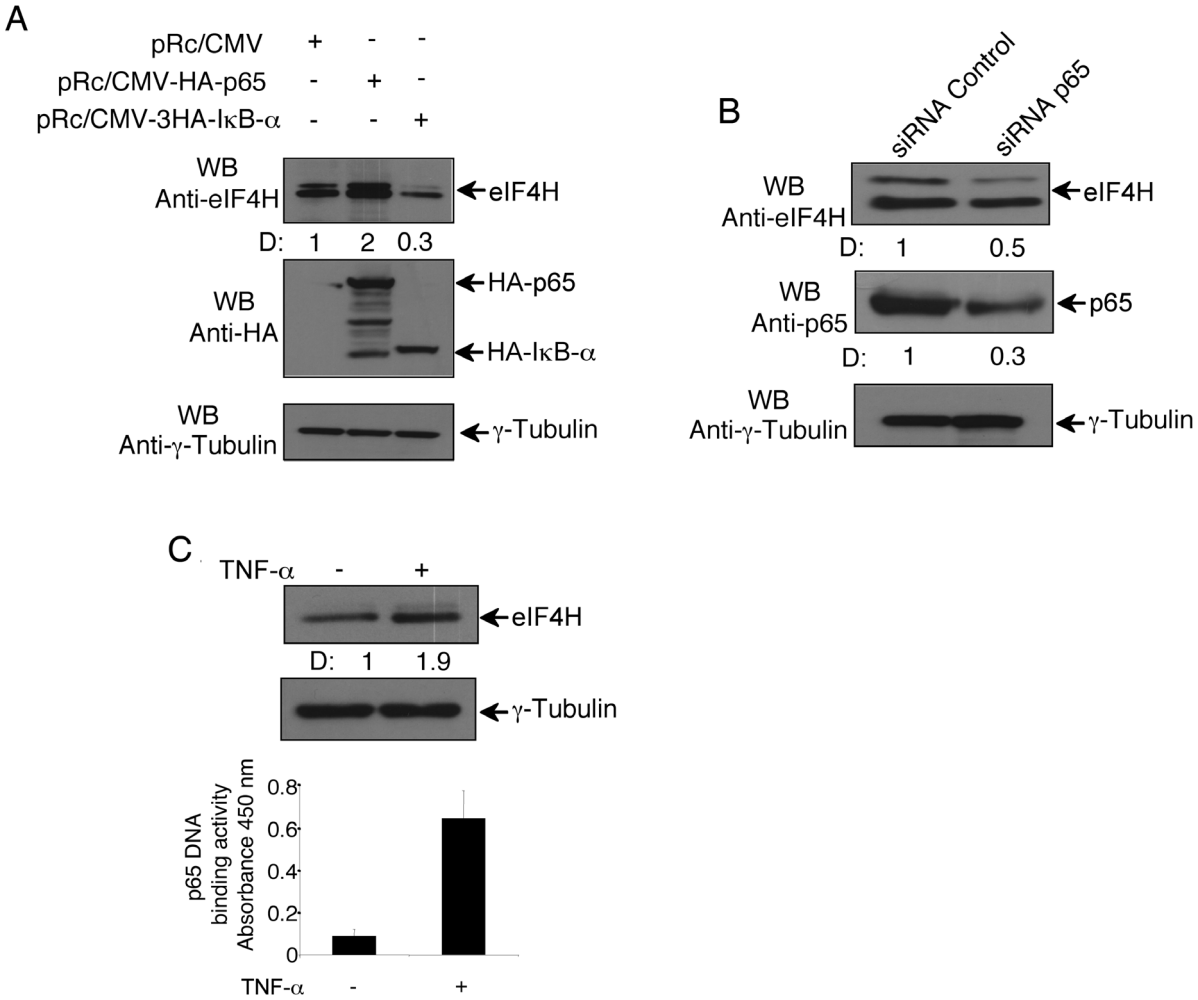
p65-dependent modulation of EIF4H protein expression. (A) HeLa cells (5×10^6^) were transfected with pRc/CMV-3HA-p65, pRc/CMV-3HA-IκB-α, or pRc/CMV empty vector (5µg), and 48h later whole cell extracts were recovered. Protein extracts (20µg) were separated by 12% SDS–PAGE and analysed by western blotting using anti-HA, anti-eIF4H, or anti-γ-Tubulin antibodies. Densitometry values (D) of the bands were expressed as fold increase above the empty vector, taken as 1. (B) HeLa cells (5×10^6^) were transfected with siRNA control, or siRNA p65 (200 pmol), and forty-eight hours post-transfection whole cell extracts were performed. Protein samples (20µg) were separated by 12% SDS–PAGE, and analysed by western blotting using anti-eIF4H, anti-p65, or anti-γ-Tubulin antibodies. Densitometry values (D) of the bands were expressed as fold increase above siRNA control, taken as 1. (C) HeLa cells (5×10^6^) were 45 min-stimulated with TNF-α (20 ng/mL), or left untreated, washed twice with DMEM, and lysed to perform total extracts and nuclear extracts. Upper panel, total cell extracts (20µg) were separated by 12% SDS–PAGE and analysed by western blotting using anti-eIF4H or anti-γ-Tubulin antibodies. Densitometry values (D) of the bands were expressed as fold increase above un-stimulated cells, taken as 1. Lower panel, nuclear extracts were analysed for the p65 binding to the NF-κB double-stranded oligonucleotide by ELISA EMSA.

Next, we asked whether the p65-dependent transcription of eIF4H affected the cellular content of eIF4H protein. To this end, HeLa cells were transfected with pRc/CMV-HA-p65, pRc/CMV-HA-IκB-α, or pRc/CMV empty vector, and forty-eight hours later the expression level of eIF4H protein was analysed in cell extracts by western blotting. As previously reported [20], two differentially spliced isoforms of eIF4H were observed in un-transfected cells showing a slower and a faster migration band on the on gel (Figure 3A). Both eIF4H isoforms were increased by p65, and decreased by the IκB-α inhibitor of NF-κB (Figure 3A), suggesting that p65 enhanced the expression of eIF4H gene without altering its splicing. Consistently, p65 RNA interference reduced the amount of both eIF4H isoforms (Figure 3B). In the same samples, γ-Tubulin, an NF-κB-independent gene, was unaffected by p65, IκB-α or p65 RNA interference (Figure 3A, B). As further analysis, TNF-α treatment significantly increased the p65 DNA binding activity as well as the eIF4H protein content (Figure 3C, upper and lower panels). Altogether these results indicated that the p65-mediated transcriptional activation of eIF4H gene resulted in increased eIF4H protein level.

### p65 Increases the Protein Synthesis Rate in eIF4H-dependent Manner

**Figure 4:**
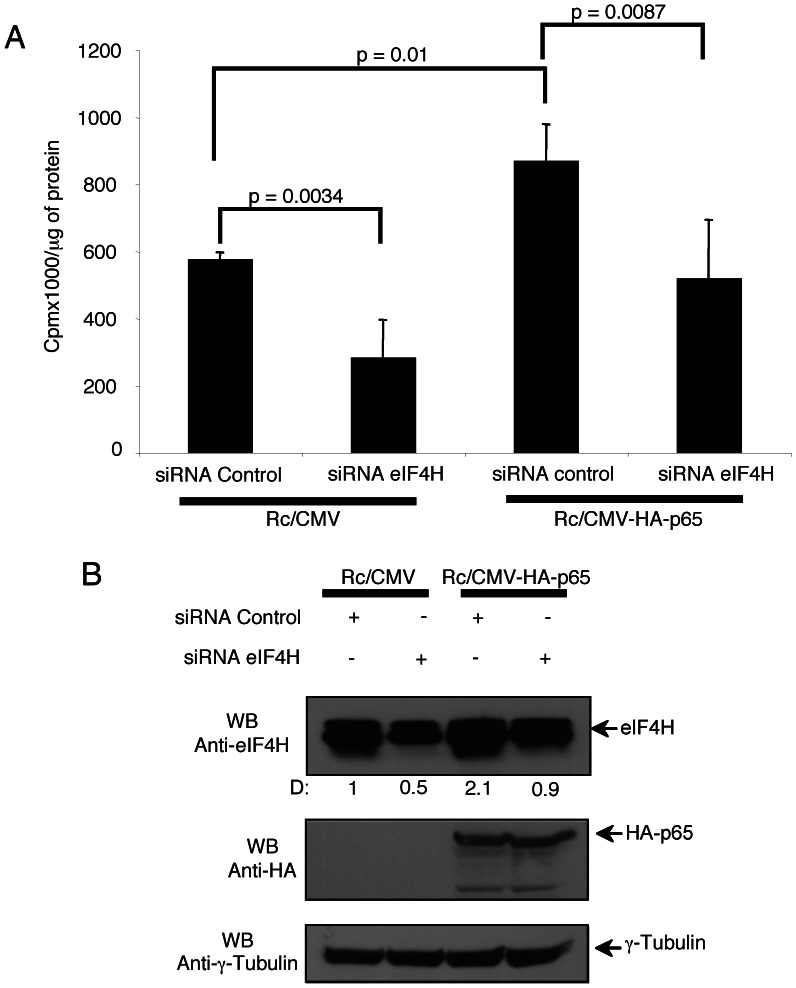
p65 increases the protein synthesis rate in eIF4H-dependent manner . (A) HeLa cells (3×10^6^) were transfected with pRc/CMV empty vector (5µg), or pRc/CMV-3HA-p65, in presence of siRNA control or siRNA p65 (200 pmol). Twenty-four hours post-transfection, cells were incubated in methionine/cysteine-free medium for 30 min before addition of labelling medium containing [^35^S]-methionine/cysteine (10 µCi/ml). One hour after protein labelling, the protein synthesis rate was evaluated. Values (mean ± SD, n = 3) are shown. Statistically significant differences between the samples are indicated according to Student's *t*-test (p≤0.01). (B) Protein extracts (20µg) of transfected HeLa cells shown in (A) were separated by 12% SDS–PAGE, and analysed by western blotting using anti-eIF4H, anti-HA, or anti-γ-Tubulin antibodies. Densitometry values (D) of the bands were expressed as fold increase above the control (Rc/CMV plus siRNA control), taken as 1.

Since eIF4H is a key regulator of translation initiation [1], [2], we tested whether p65 could affect the global protein synthesis by up-regulating the cellular content of eIF4H. HeLa cells, which had been silenced for eIF4H or left unsilenced, were transfected with pRc/CMV-p65, or pRc/CMV empty vector, and forty-eight hours later the translation rate was determined by [^35^S] methionine/cysteine metabolic labelling. The protein synthesis rate as well as the eIF4H content was significantly increased in p65-transfected cells as compared to empty vector (Figure 4A, B). Moreover, eIF4H RNA interference halved the protein synthesis rate in empty vector-transfected cells, and abolished the enhancement of protein synthesis in p65-transfected cells (Figure 4A, B), indicating a strict correlation between the eIF4H expression and the rate of protein synthesis. These results suggest that p65 promoted the global protein synthesis by up-regulating eIF4H.

## Materials and Methods

### Plasmids, Cell Transfection and Treatments

Plasmids pRc/CMV, pRc/CMV-HA-p65, pRc/CMV-p50 and pRc/CMV-HA-IκB-α were previously described [21]–[23]. pMT2T-RelB and pMT2T-c-Rel were kindly provided by Dr. Antonio Leonardi, University of Naples “Federico II”, Naples, Italy. Wild type and p65^−/−^ MEFs were kindly provided by Dr. A. A. Beg, Moffitt Cancer Center, Department of Immunology, Tampa, FL 33612, USA. MDA-MB-231, MCF-7, SH-SY5Y, U251, D54 tumor cell lines were purchased from the American Type Culture Collection, Manassas, VA, USA. HeLa, MDA-MB-231, MCF-7 and MEFs were cultured in Dulbecco's modified Eagle's medium supplemented with 10% heat-inactivated foetal calf serum and 2 mM L-glutamine (Lonza Cologne AG, Germany), while SH-SY5Y, U251 and D54 cells were cultured in Dulbecco's modified Eagle's medium/Ham’s F12 (1∶1 ratio) supplemented with 10% heat-inactivated foetal calf serum, 2 mM L-glutamine, 1 mM sodium pyruvate and 0.1 mM non-essential amino-acids. MC3 and DeFew cells were cultured in RPMI1640 (Lonza Cologne AG, Germany) supplemented with 10% heat-inactivated foetal calf serum and 2 mM L-glutamine.

Cells were transfected with DNA or siRNA by using FuGENE HD (Roche Diagnostic GmbH, Mannheim, Germany), according to the manufacturer's protocol. RNA interference of p65 and eIF4H was performed with SMART pool siRNA p65 and eIF4H (Dharmacon, Chicago, IL, USA), respectively. Transfection of small interfering RNA was performed as previously described [24], [25].

HeLa cells were treated with TNF-α (20 ng/ml, Sigma-Aldrich, St Louis, MO, USA) for 45 min, washed twice with complete culture medium, and lysed in lysis buffer using Dual Light Luciferase System (Tropix, Bedford, MA, USA).

### Cell Extracts and Western Blotting

Total cell extracts and western blotting analysis were performed as previously described [26], [27]. Briefly, protein aliquots were resuspended in loading buffer (125 mM Tris–HCl, pH 6.8, 5% SDS, 1% bromophenol blue, 10% β-mercaptoethanol, 25% glycerol), resolved on 12% SDS–PAGE, transferred to polyvinylidene difluoride membrane (Millipore, Bedford, MA, USA), and incubated with primary antibodies (1∶1000), followed by incubation with horseradish-peroxidase-linked mouse or rabbit IgG (1∶2000) (GE Healthcare Amersham, Little Chalfont, Buckinghamshire, UK) in PBS containing 5% non-fat dry milk (Bio-Rad Laboratories). Proteins were detected by chemiluminescence using the ECL System (GE Healthcare Amersham). Primary antibodies were purchased from Santa Cruz Biotechnology, Santa Cruz, CA, USA (anti-HA F7); Sigma-Aldrich (anti-γ-Tubulin); Upstate, Lake Placid, NY, USA (anti-p65). Densitometry of single bands in western blots was performed by ImageJ software package (NIH, USA).

### NF-κB DNA Binding Assay

The p65 binding to the double-stranded NF-κB oligonucleotide was measured by ELISA EMSA using the NF-κB Combo Transcription Factor Assay kit (Cayman Chemical Company, Ann Arbor, MI, USA), as previously described [28].

### qRT-PCR

RNA extraction and qRT-PCR were performed as previously described [29], [30]. Briefly, RNA aliquots (1 µg) were reverse transcribed using Random Examers (Roche) and Superscript III Reverse Transcriptase (Invitrogen), according to the manufacturer's protocol. qRT-PCR was carried out with the iCycler iQ Real-Time detection system (Bio-Rad Laboratories) under the following conditions: 95°C, 1 min; (94°C, 10s; 60°C, 30s) ×40. Primers were as follows: eIF2S2, 5′- TGCAGAAGGACACACGACTC-3′ and 5′-AACCTGTCCAGCCACATCTC-3′; eIF2S3, 5′-CGTCAGGATCTCACCACCTT-3′ and 5′-ACTGTGGATTTCCCATGAGC-3′; eIF3E, 5′-GACTTTGATGGGGCTCAGAA-3′ and 5′-ATACACTGGTGGATGCGACA-3′; eIF4A1, 5′-GTGCAGAAACTGCAGATGGA-3′ and 5′-TGTTGCTGTTGAGCTTTTGG-3′; eIF4A2, 5′- GATGCATGCCAGAGACTTCA-3′ and 5′-AACACGACTTGACCCTGACC-3′; eIF4G2, 5′-GACAGAAGCAAAGCACCACA-3′, 5′-GCAATGGCTCTCTGTTCCTC-3′; eIF4H, 5′-TGGCTTCAGGGATGACTTCT-3′ and 5′-CGAGGTTTAAGCTGGAGTCG-3′; eIF5B, 5′- GATGGCATGGGAAGTCTGAT-3′ and 5′-CTCCATCACCTGTGCTCTCA-3′; eIF2B3, 5′-CCGGAGTGAACTGATTCCAT-3′ and 5′- ATTCCAGCAGGCATCATAGG-3′. Gene expression levels were calculated relative to GAPDH mRNA levels as endogenous control. mRNA Relative Expression was calculated as 2^∧^(Ct ^gene under investigation^ − Ct ^GAPDH^).

### ChIP Assay

HeLa cells were fixed by adding formaldehyde (Sigma-Aldrich) to the final concentration of 1% and after 10 min incubation, ice-cold PBS plus 0.125 M glycine was added; plates were transferred on ice, washed extensively with PBS, and scraped. Upon centrifugation, cells were 10 min-lysed in lysis buffer (5 mM PIPES pH 8.0, 85 mM KCl, 0.5% NP-40) supplemented with 1× Complete Protease Inhibitor (Roche Diagnostic GmbH). Nuclei were collected by centrifugation (1000 × *g*, 5 min), and resuspended in sonication buffer (50 mM Tris–HCl pH 8.0, 1% SDS, 10 mM EDTA). Chromatin was sonicated using Bandelin Sonoplus GM70 (Bandelin Electronic, Berlin, Germany), centrifuged (14,000×*g*, 15 min), and supernatant was 10-fold diluted in dilution buffer (0.01% SDS, 16.7 mM Tris–HCl pH 8.0, 1.1% Triton X-100, 167 mM NaCl, 1.2 mM EDTA). Samples were pre-cleared by 3h-incubation with 20µl of protein G agarose beads, followed by incubation with antibodies against the analysed proteins. The antibodies anti-p65 (sc-372), anti-p50 (sc-1190), anti-RelB (sc-226), anti-c-Rel (sc-71) and rabbit IgG (sc-2027) were from Santa Cruz Biotechnology. Immunoprecipitations were carried out overnight at 4°C and immune complexes were collected with protein G agarose beads, washed five times with low salt buffer (20 mM Tris–HCl pH 8.0, 0.1% SDS, 1% Triton X-100, 2 mM EDTA, 150 mM NaCl), four times with high salt buffer (20 mM Tris-HCl pH 8.0, 0.1% SDS, 1% Triton X-100, 2 mM EDTA, 500 mM NaCl), once with TE buffer (10 mM Tris–HCl pH 8.0, 1 mM EDTA), and extracted in TE buffer containing 2% SDS. Protein-DNA cross-links were reverted by heating at 65°C overnight. DNA was further purified by QIAquick PCR purification kit (QIAGEN) and eluted in 50µl sterile distilled water. Specific enrichment in NF-κB enhancer sequences was measured by real-time PCR of ChIP eluates using SYBR GreenER Master Mix (Invitrogen). Reactions were carried out with the iCycler iQ Real-Time detection system (Bio-Rad Laboratories) using the following conditions: 95°C, 1 min; (94°C, 10s; 60°C, 30s) ×40. For each sample, values were normalized to input DNA and reported as % of input over the rabbit IgG control. Primers were as follows: eIF4H, 5′-ATGTATGGAGGCCGAAAGG-3′ and 5′-CTACGCGGCCCATTATGT-3′; eIF2S3, 5′-AAGAGCCCCGTCATAGGTG-3′ and 5′-GGGAGAGACCATTTCCGTTT-3′.

### Protein Synthesis Rate Analysis

HeLa cells (3×10^6^) were incubated in methionine/cysteine-free medium for 30 min before addition of labelling medium containing [^35^S]-methionine/cysteine (10 µCi/ml) (EasyTag Express Protein Labelling Mix, [^35^S], Perkin Elmer, Boston, MA, USA). One hour post-protein labelling, cells were harvested, washed twice in PBS, and lysed in lysis solution (Tropix, Bedford, MA, USA). Protein samples (10 µg) were loaded on 3MM paper and precipitated by sequential incubations in cold and boiling 10% trichloroacetic acid (TCA) (5 min per step), and briefly washed in acetone and ethanol. Filters were air-dried, and incorporation of [^35^S]-methionine/cysteine was measured by liquid scintillation counting.

### Statistical Analysis

Statistical analysis was performed by two-tail unpaired Student’s *t*-test. Data were reported as means ± SE. Differences between the means were considered as statistically significant at the 99% level (*p*≤0.01).

## Discussion

Gene expression and translation regulation must be coupled in order to make available specific proteins in response to signalling. As first step of protein synthesis, translation initiation is highly and timely regulated by the eIFs activity. In this study, we have shown that the eIF4H gene contains two tandem NF-κB binding sites in the proximal promoter, and it was transcriptionally activated by the p65 subunit of NF-κB. RNA interference of p65 decreased the basal expression level of eIF4H, indicating that endogenous p65 regulated the basal expression of eIF4H. Of interest, p65 did not affect the expression of other eIFs containing putative NF-κB enhancers in their promoters, indicating that, among the analysed genes, eIF4H was the only one to be subjected to the transcriptional control of p65. These findings suggested a regulatory mechanism of protein synthesis mediated by eIF4H in response to NF-κB signalling. Based on this hypothesis, NF-κB activation signalling was expected to increase the eIF4H protein content in order to stimulate the initiation translation, as a coordinate action between the NF-κB-dependent gene expression and protein synthesis, being eIF4H a key regulatory sensor. Accordingly with this hypothesis, we found that TNF-α, a well-characterized activator of NF-κB, increased both the transcription and protein content of eIF4H, being such effect dependent on p65 activity and p65 recruitment to the eIF4H promoter. Furthermore, the global protein synthesis rate correlated with the p65 activity and eIF4H expression, being the effect of p65 on protein synthesis mediated by eIF4H.

eIF4H regulates the activity of eIF4A, which is the best-characterized RNA helicase that unwinds secondary structures at the 5′-untranslated region in translation initiation [2]. eIF4H associates with eIF4A in order to increase the binding affinity of eIF4A for ATP [4] and RNA [31], [32], thus promoting the processivity of eIF4A [3], [33]–[36]. Little is known on eIF4H regulation by signalling. In this regard, the evidence of a transcriptional control of eIF4H exerted by NF-κB provides new insights into regulation of eIF4H-dependent translation in response to NF-κB-activation signalling.

NF-κB transacting factors promote cell survival by activating the transcription of growth factors, anti-apoptotic genes, positive cell-cycle regulators, and by antagonizing the transcriptional activity of tumour suppressor p53 [37]. Enhanced NF-κB activity was associated with development of tumorigenic phenotype [37]. Indeed, NF-κB inhibitors, including the super-repressor IκB-α S32/36A and proteasome inhibitors, counteracted the tumour growth [18]. Based on the evidence of NF-κB-dependent regulation of eIF4H gene expression, it is reasonable to hypothesize that constitutively active NF-κB could up-regulate protein synthesis by enhancing the expression of eIF4H. In this regard, the expression of a spliced isoform of eIF4H was shown to be strictly associated with the tumour behaviour and increased translation rate of peculiarly structured 5′cap mRNAs, including those of proliferative functions, such as cyclin D1 [20]. A similar evidence was reported for eIF4B, the human paralogue of eIF4H, which promoted the tumour growth and increased the translation of mRNAs harbouring 5′UTR secondary structures, including oncogenes [38]. Consistently with these previous observations, we found a strict correlation between p65/NF-κB activity and eIF4H expression levels in a panel of tumour cell lines, suggesting that the sustained NF-κB activity in these cells could promote cell growth by up-regulating the eIF4H-dependent translation initiation. The evidence of NF-κB-dependent transcriptional control of eIF4H could expand the spectrum of action of NF-κB inhibitors in chemotherapy as these agents could inhibit the global protein synthesis by repressing the eIF4H gene expression.

## Supporting Information

Figure S1 Analysis of effect of NF-κB family members on eIF genes expression. HeLa cells (5×10^6^) were transfected with pRc/CMV-p50, pMT2T-RelB, pMT2T-c-Rel or pRc/CMV-empty vector (5 µg). Forty-eight hours post-transfection, total RNA was extracted and analysed by qRT-PCR to measure the expression of the indicated eIF genes. Values (mean ± SD, n = 3) are shown.
(TIF)
