# Supplementary figures and images for: Eukaryotic Initiation Factor 4H Is under Transcriptional Control of p65/NF-κB
Source: PLoS One. 2013 Jun 11;8(6):e66087. doi: 10.1371/journal.pone.0066087 (PMC3679033; doi:10.1371/journal.pone.0066087)

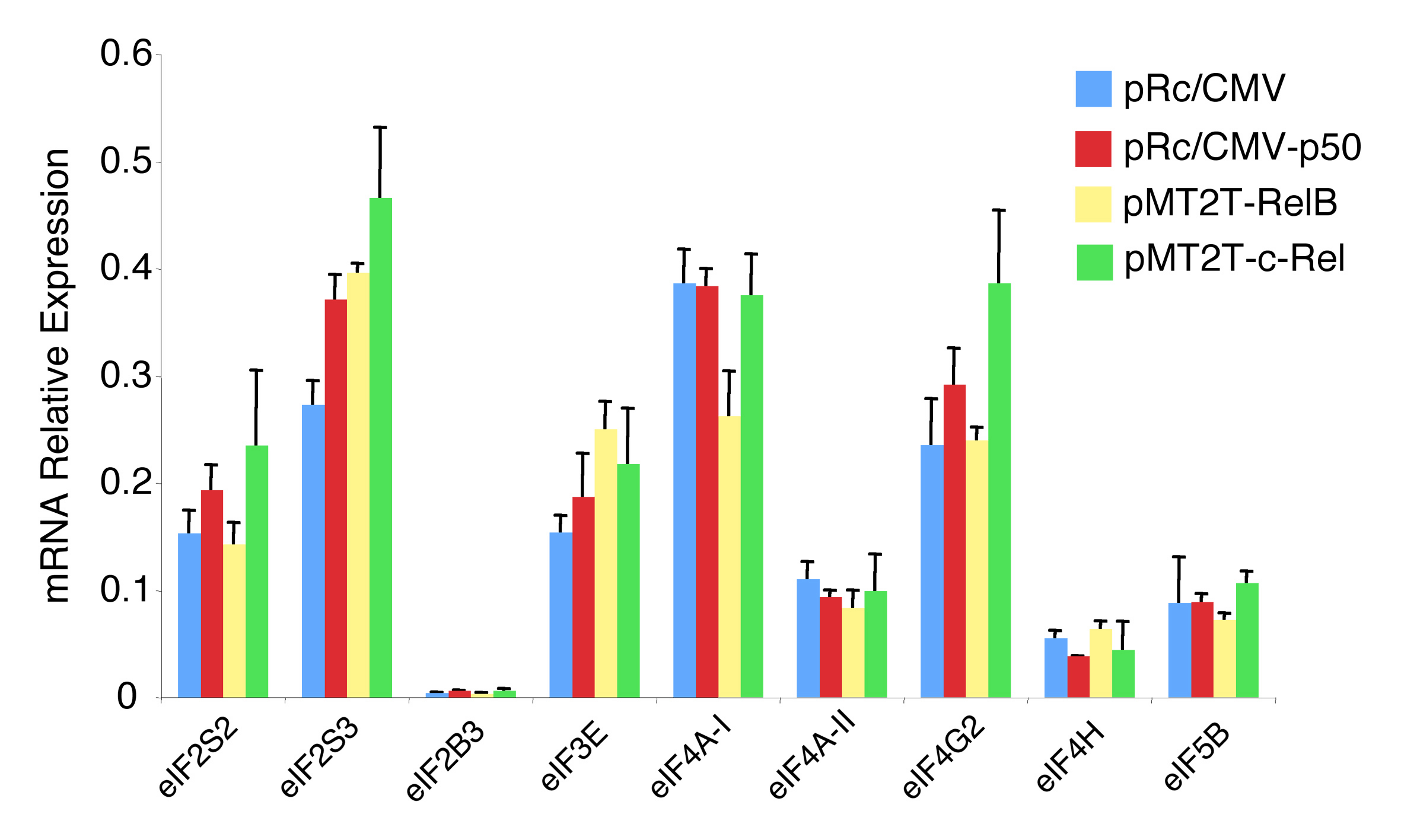

Supplement: Figure S1 — Analysis of effect of NF-κB family members on eIF genes expression. HeLa cells (5×10^6^) were transfected with pRc/CMV-p50, pMT2T-RelB, pMT2T-c-Rel or pRc/CMV-empty vector (5 µg). Forty-eight hours post-transfection, total RNA was extracted and analysed by qRT-PCR to measure the expression of the indicated eIF genes. Values (mean ± SD, n = 3) are shown. (TIF) [file pone.0066087.s001.tif]
